# Antibacterial performance of a porous Cu-bearing titanium alloy by laser additive manufacturing

**DOI:** 10.3389/fbioe.2023.1226745

**Published:** 2023-08-03

**Authors:** Jiawei Xu, Yanjin Lu, Xiyun Pan, Desong Zhan, Qiang Wang, Ning Zhang

**Affiliations:** ^1^ School and Hospital of Stomatology, China Medical University, Shenyang, China; ^2^ Liaoning Provincial Key Laboratory of Oral Diseases, Shenyang, China; ^3^ Key Laboratory of Optoelectronic Materials Chemistry and Physics, Fujian Institute of Research on the Structure of Matter, Chinese Academy of Sciences, Fuzhou, China

**Keywords:** laser additive manufacturing, porous titanium alloy, copper, antibacterial property, bacterial biofilm, Porphyromonas gingivalis

## Abstract

*Porphyromonas gingivalis (P. gingivalis)* is the most common species that causes peri-implantitis. It forms an irreversible dense biofilm and causes inflammation. A novel 3D-printed porous TC4-6Cu alloy was fabricated using selective laser melting (SLM) technology for the dental implant, which is anticipated to inhibit biofilm formation. We attempted to investigate the antibacterial ability and antibacterial mechanism of the 3D-printed porous TC4-6Cu alloy against *P. gingivalis*. This work used scanning electron microscopy (SEM) and laser confocal microscopy (CLSM) to detect the antimicrobial ability of the alloy against sessile *P. gingivalis*. The results indicated that the 3D-printed porous TC4-6Cu alloy could cause bacterial fragmentation and deformation. Plate antimicrobial counting experiments showed that the antibacterial rates of the alloy against adherent bacteria and planktonic bacteria after 24 h were 98.05% and 73.92%, respectively. The minimum inhibitory concentration (MIC) and minimum bactericidal concentration (MBC) of Cu^2+^ were tested to appraise the antibacterial property of the alloy against planktonic *P. gingivalis.* The relationship between the antibacterial mechanism of the alloy with oxidative stress was evaluated through ROS fluorescence intensity and protein leakage concentration. The results revealed that the alloy significantly eliminated adherent bacteria and inhibited biofilm formation. Moreover, 3D-printed porous TC4-6Cu alloy demonstrated significant bactericidal ability by inducing the production of reactive oxygen species (ROS), which could result in protein leakage from the bacterial cell membrane. This research may open a new perspective on the development and biomedical applications for dental implantation.

## 1 Introduction

The number of patients requiring the replacement of failed tissue with artificial implants, including craniofacial and maxillary implants, has recently increased (Geng et al., 2017). The implanted biomaterials determine the success of dental and orthopedic procedures (Sarraf et al., 2022). Dental implantation can restore the function and aesthetics of missing teeth. Studies indicate that approximately 300,000 individuals annually undergo dental implant surgery (Wang et al., 2015). Titanium alloys are the most commonly used implant materials in the field of stomatology (E. P. Su et al., 2018) due to their excellent mechanical and chemical properties, good biocompatibility, as well as capacity to resist corrosion (Liu et al., 2021). However, titanium alloys are biologically inert. Bacteria adhere to the surface of the implant, causing biomaterial-centered infection (BCI), resulting in surgical failure (Khodaei and Hossein Kelishadi, 2018). *Porphyromonas gingivalis (P. gingivalis)* primarily causes peri-implantitis, which is an inflammatory lesion of soft and hard tissues around the dental implant. *Porphyromonas gingivalis* can express numerous virulence factors, including fimbriae (Hasegawa and Nagano, 2021), gingipains, and hemagglutinin (Sundaram et al., 2019), establishing itself under the gum, resulting in tissue destruction and peri-implantitis (Xu et al., 2020). *Porphyromonas gingivalis* has multiple mechanisms for survival under stressful conditions (Song et al., 2022) forming a biofilm to destroy adjacent tissues, resulting in the loosening and dislocation of dental implants (Zhu et al., 2015).

At present, antibiotics are administered to improve the antimicrobial properties of implant materials (Jahanmard et al., 2020). However, systemic antibiotic therapy as part of the treatment regimen for peri-implantitis faces hurdles, including the development of antibiotic resistance (Salgado-Peralvo et al., 2021). In the United States alone, the direct economic cost of antimicrobial-resistant (AMR) infections exceeds $20 billion a year (de Kraker et al., 2016). Therefore, developing dental implants with antibacterial functions is necessary to prevent infection preoperatively and perioperatively.

Researchers have developed monolithic antimicrobial materials (Liu et al., 2020a), most commonly by adding metals, including copper and silver, to improve their antimicrobial properties (van Hengel et al., 2020). However, silver is toxic to the tissue (Amin Yavari et al., 2016) with insignificant antimicrobial effects in clinically relevant bone models. Zhen Geng et al. discovered that Ag incorporation in the HA coating revealed significant antibacterial efficacy, however, the cell viability sharply decreased due to Ag cytotoxicity (Geng et al., 2016). Copper is a vital metal element in metabolizing cholesterol, glucose, and amino acids (Xia et al., 2020). In 2008, the U.S. Environmental Protection Agency (USEPA) declared copper as an effective metal antimicrobial agent that could directly kill bacteria through “contact killing” (Vincent et al., 2018).

Similar to other metal ions, the precise antibacterial mechanism of copper ions remains unclear (Wang et al., 2021). An imbalance of oxidation and antioxidation in and outside the bacterial microorganism induces oxidative stress, generating several oxidation products (ROS). High ROS causes oxidative damage to key molecules, including proteins, lipids, and DNA, within bacterial microorganisms. Previous studies suggest that the mechanisms by which copper ions induce ROS production include: a) Copper ions can disrupt protein function by catalyzing the oxidation of sensitive amino acids within bacterial cells, eventually degrading them (Duan et al., 2022); b) Copper ions induce ROS to produce free oxygen radicals, causing peroxidative damage of lipids in the bacterial cell membranes, hence reducing membrane fluidity. Consequently, the membrane ruptures, causing bacterial death (Godoy-Gallardo et al., 2021); c) Copper ions can function through the Fenton reaction, converting hydrogen peroxide into highly toxic hydroxyl radicals, which destroy the donors and ligands of bacterial cells. Copper ions can disrupt the electron transport process during bacterial metabolism and promote ROS production (Goudouri et al., 2014); d) Copper ions can directly damage bacterial DNA by inducing ROS production (Liang et al., 2020). In summary, copper ions have antibacterial properties and could be added to implant materials. Therefore, the specific antibacterial mechanism of copper ions warrants clarification.

Selective laser melting technology (SLM) is the preparation of materials by selectively melting metal powders using high-power lasers (Yuan et al., 2019). SLM enables the production of biomedical implants with individualized porous structures to obtain mechanical properties similar to human bones and prevent stress shielding effects (Taniguchi et al., 2016). Additionally, SLM significantly improves the effective antimicrobial surface area of porous materials (Pałka and Pokrowiecki, 2018). The essence of additive manufacturing technology is the layer-by-layer stacking and melting of metal powder. Additionally, powder particles must be on the surface of the molded material that has not been completely melted. It is easy to gradually peel off these powder particles due to micro-motion wear to form wear particles, inducing the occurrence of peri-implantitis (Yi et al., 2022). Acid etching is broadly used in commercial dental implants, including Straumann^®^ Ti SLA^®^ dental implants and Dentply implants. Acid etching is performed by soaking commercial dental implants in strong acids (Yan Guo et al., 2012), including hydrochloric, nitric, hydrofluoric, and sulfuric acids, to remove contaminants from the implant surface (Cura et al., 2022).

In the present study, a 3D-printed porous TC4-6Cu alloy was fabricated by additive manufacturing technology. The 3D-printed porous TC4-6Cu alloy was selectively acid-etched to remove un-melted metal particles on its surface. The antibacterial ability and antibacterial mechanism of the 3D-printed porous TC4-6Cu alloy against *P. gingivalis* were investigated. This study aims to provide a reference for the clinical application of the 3D-printed porous TC4-6Cu alloy.

## 2 Materials and methods

### 2.1 Sample preparation

The 3D-printed porous TC4-6Cu alloy was fabricated by the Institute of Metal Research, Chinese Academy of Sciences. Commercial Ti6AI4V powder was prepared into porous and solid TC4 alloy using the SLM technology. The commercial Ti6AI4V and copper powders were mixed to prepare porous and solid TC4-6Cu alloy. The physical dimension was 10 mm × 10 mm × 2 mm. The materials were then polished using 800 mesh, 1000 mesh, 1500 mesh, and 2000 mesh sandpaper. The specimens were subsequently acid-etched in diluted nitric acid and hydrofluoric acid solution for 20 s. Thereafter, the specimens were rinsed with running water for 1 min. Eventually, all materials were successively subjected to 10 min of ultrasonic cleaning in acetone, distilled water, and absolute ethanol. After drying the materials at room temperature, they were sterilized by autoclaving at high temperatures. The selectively acid-etched materials included TC4-SAE, TC4-6Cu-SAE, Porous-TC4-SAE, and Porous-TC4-6Cu-SAE. In contrast, TC4, TC4-6Cu, Porous-TC4, and Porous-TC4-6Cu were not treated with selective acid etching. TC4 was the control material.

### 2.2 Microstructure and element composition

The macroscopic structure of materials was observed using Micro-CT. The microstructure was observed using SEM (SEM, Zeiss Merlin Compact, Zeiss, Germany). The elemental composition of materials was determined using the energy-dispersive spectrometer (Zeiss Merlin Compact, Zeiss, Germany).

### 2.3 Anaerobic bacteria culture and medium

*Porphyromonas gingivalis* standard strains ATCC 33,277 (provided by Department of Oral Biology, School and Hospital of Stomatology, China Medical University, Shenyang) were removed from the frozen glycerol and BHI liquid medium mixture (BHI, pH = 7.0), passaged onto BHI solid blood medium (Meilun, China), and re-passaged after 5–7 days through incubation at 37°C under anaerobic conditions (90% N_2_ and 10% CO_2_). After speciation, the bacteria were cultured on a solid medium for another 48 h under similar conditions before used in further experiments.

### 2.4 SEM observation of sessile *Porphyromonas gingivalis*

The morphology of adherent bacteria on the surface of the materials was observed using scanning electron microscopy. After incubating the materials and *P. gingivalis* for 24 h and 72 h according to the above experimental methods, the planktonic bacteria on the surface of the materials were gently washed off with PBS. The materials were soaked in 2.5% glutaraldehyde solution at 4°C for 4 h to fix the bacterial morphology. After the fixation, the materials were washed with PBS, followed by gradient dehydration in varying concentrations of ethanol solution (50%, 60%, 70%, 80%, 90%, 95%, and 100%) for 10 min. The materials were coated with gold after drying at room temperature.

### 2.5 Quantitative analysis of the antibacterial ability of materials

The antibacterial capacity of materials against *P. gingivalis* was determined using plate colony counting. The materials were placed in a 24-well plate. Next, 1 mL of bacterial suspension (1 × 10^7^ CFU/mL) was added to each well and incubated at 37°C for 24 h and 72 h under anaerobic conditions to establish a blank bacterial control group without materials.

#### 2.5.1 Antibacterial action on sessile P. gingivalis

At the established time point, after removing the materials and gently washing them with sterile PBS, each material was placed in a 10 mL EP tube containing 1 mL PBS and vortexed for 1 min. The obtained bacterial adhesion suspension was serially diluted with PBS to a final concentration of 1 × 10^5^ CFU/mL. A total of 100 μL bacterial suspension was evenly coated on BHI blood plates. Finally, colony counting was performed, and the results were photographed after incubation at 37°C for 7 days under anaerobic conditions. The antibacterial rate was calculated as follows:

$$R=\left({CFU}_{contrast\;group}–\;{CFU}_{experimental\;group}\right)/{CFU}_{contrast\;group}\times 100\%$$

Where R is the antimicrobial rate, and CFU is the number of colony-forming units.

R ≥ 99% indicated that the material had a strong bactericidal effect, while R ≥ 90% indicated that the material had a bactericidal effect.

#### 2.5.2 Antibacterial action on planktonic *Porphyromonas gingivalis* and pH determination

At the set time point, we collected the bacterial suspension co-cultured with the materials in a 1.5 mL EP tube, mediated, and shook for 1 min. The coating experimental method and calculation method were performed as described in section 2.5.1. At the same time, the pH of the co-cultured bacterial suspension was determined using a pH meter (FE28-FiveEasy PlusTM, METTLER TOLEDO).

### 2.6 Crystal violet stain

The antibacterial biofilm effect of the materials was quantified through a crystal violet staining experiment. After incubation for 24 h and 72 h, the plankton bacteria on the surface of the materials were washed off with PBS, placed in methanol for fixation, and dried at room temperature for 30 min (after 15 min). After staining the material with 0.1% crystal violet for 10 min, the excess dye was washed off and dried at room temperature. The dried materials were decolorized with absolute ethanol, and the eluted stain was placed in a 96-well plate. The absorbance of the materials were measured at 600 nm using a microplate reader (Tecan, Salzburg, Austria).

### 2.7 Bacterial viability analysis

The bacterial activity was assessed by performing live-death staining of materials co-cultured with bacterial suspension for 24 h and 72 h using the live/dead bacterial viability kit (Invitrogen-Eugene, USA). The co-cultured materials were removed, and the unadhered bacteria were gently washed off with PBS. The materials were stained for 20 min in the dark. After staining, the fluorescently stained bacteria images were captured using laser confocal microscopy (CLSM, Olympus FV3000, Japan). SYTO-9 marked surviving bacteria in green, whereas PI marked dead bacteria in red.

### 2.8 Qualitative analysis of intracellular ROS of *Porphyromonas gingivalis*

The ROS generated in the cells of *P. gingivalis* co-cultured with the materials was qualitatively detected using the Intracellular ROS Assay Kit (Beyotime Biotechnology Ltd., Shanghai, China). After incubating the materials and *P. gingivalis* suspension for 6 h, 24 h, and 72 h based on the above experimental methods, the planktonic bacteria were removed by PBS. In the 24-well plate, we added 500 μL of BHI liquid medium containing 10 μM 2′, 7′-Dichlorodihydrofluorescein-diacetate probe (DCFH-DA) to each well and incubated at 37°C for 20 min to load the fluorescent probe into the bacteria. The materials were protected from light throughout the experiment. Fluorescently stained bacteria were captured and observed under an inverted fluorescence microscope (Nikon Corporation, Japan).

### 2.9 Protein leakage assay

The permeability of the membrane and cell wall of *P. gingivalis* can be reflected by the leakage of protein within it. The concentration of the extracellular protein of the bacteria was quantified using the BCA Protein Detection Kit (Beyotime, China). The bacteria and materials were cultured for 24 h and 72 h according to the above experimental methods. After reaching the established time point, the plankton bacteria were removed by PBS. The bacteria on the materials and in the medium were collected. The standard protein and BCA working solutions were prepared following the manufacturer’s instructions. On the 96-well plate, we set up 3 sub-wells for each material. The specimens were incubated at 37°C for 20 min. The absorbance was measured at 562 nm using a microplate reader (Tecan, Salzburg, Austria). The bacteria-free BHI medium was set up as a blank control group. The experiment was replicated three times to obtain the protein concentration based on the standard protein curve and the sample volume used.

### 2.10 The determination of MIC and MBC

The improved broth dilution method required by the Clinical and Laboratory Standards Institute (CLSI) was performed to determine the MIC and MBC of Cu^2+^. After dilution, the gradient concentration of copper ion was set up, and two parallel experimental groups were established for each gradient concentration. In a 24-well plate, 500 μL of *P. gingivalis* suspension and CuCl_2_ BHI medium solution were added to each well. Then, they were mixed homogeneously to a concentration of 1 × 10^7^ CFU/mL. The MIC value was recorded after the plate was incubated at 37°C for 24 h under anaerobic conditions. The mixture was transferred to a 1.5 mL EP tube and vortexed. Thereafter, 100 μL of liquid from each tube was picked and uniformly coated on BHI solid amniotic blood medium. After incubating under similar conditions for 72 h, we took pictures to determine the MBC value.

### 2.11 Statistical analysis

All experiments were replicated at least three times. All statistical analyses were performed using GraphPad Prism software version 9.4.1 (GraphPad Software, San Diego, California, United States). Data were analyzed using a *t*-test and one-way analysis of variance (ANOVA). Continuous normally distributed data were expressed as mean ± standard deviation (SD). *p* = 0.05 was considered statistically significant.

## 3 Results

### 3.1 Microstructure and surface properties of the materials

**FIGURE 1:**
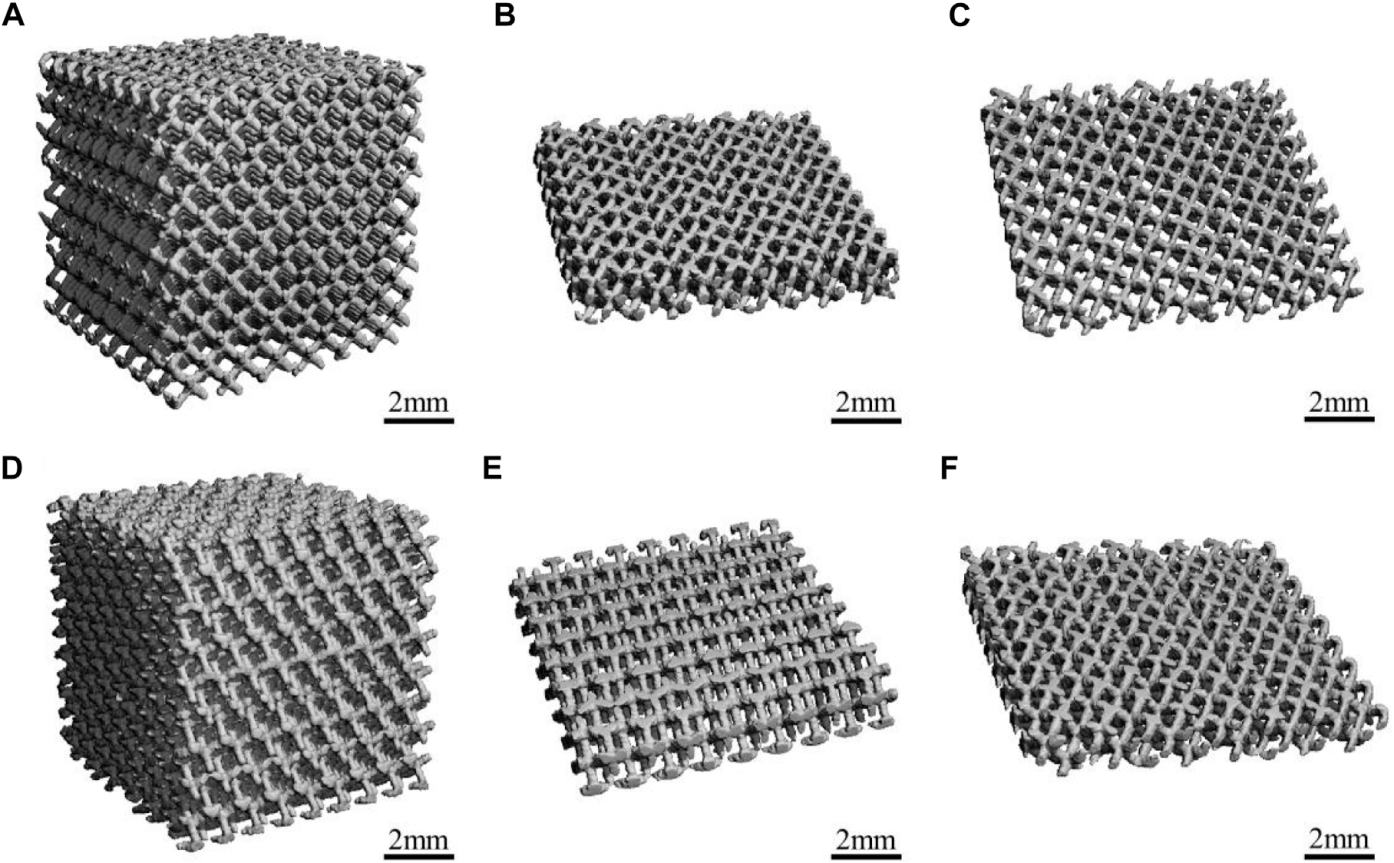
The Micro-CT diagrams of the materials: **(A,B)** Porous-TC4; **(C)** Porous-TC4-SAE; **(D,E)** Porous-TC4-6Cu; **(F)** Porous-TC4-6Cu-SAE (SAE: selective acid etching).

Figure 1 presents a macroscopic image of the materials captured using Micro-CT, all of which have similar 3D porous structures and uniform pore size. As shown in Figure 1, the structure and pore size of the materials were not changed after the acid etching treatment.

**FIGURE 2:**
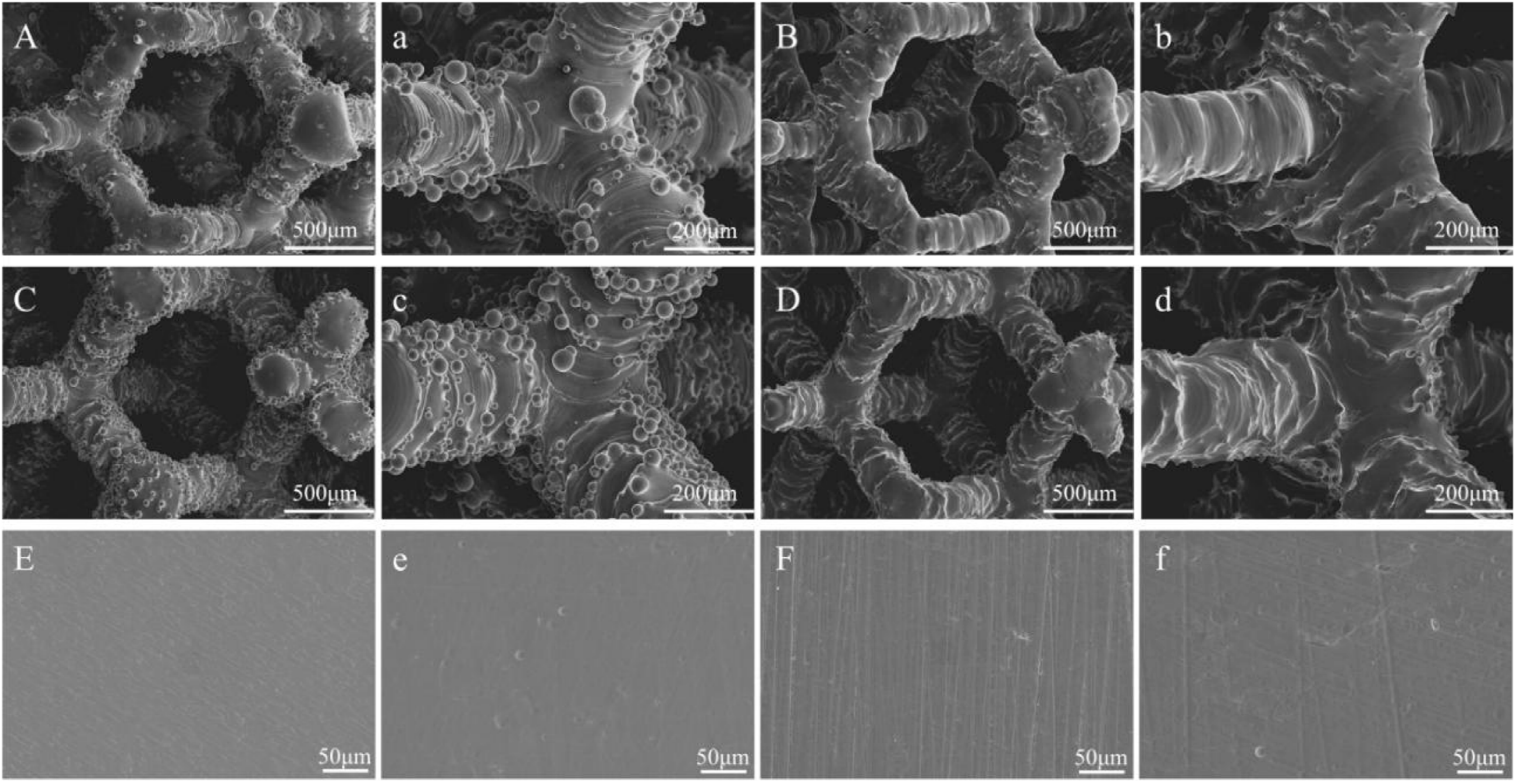
The SEM images of the materials: **(A,a)** Porous-TC4; **(B,b)** Porous-TC4-SAE; **(C,c)** Porous-TC4-6Cu; **(D,d)** Porous-TC4-6Cu-SAE; **(E)** TC4; **(e)** TC4-SAE; **(F)** TC4-6Cu; **(f)** TC4-6Cu-SAE (SAE: selective acid etching).

Figure 2 shows the microscopic morphology of the materials captured using SEM. SEM analysis showed that all porous materials subjected to acid etching had a layer of unmelted metal powder attached to the surface, which varied in size with uneven distribution. In line with previous results, the metal powder on the surface of the porous materials was completely removed after acid etching. Additionally, the structure of the materials remained unaffected. After etching the solid materials, no significant change was observed in the surface of the materials. Only a few small pits were scattered on the surface of the materials.

**FIGURE 3:**
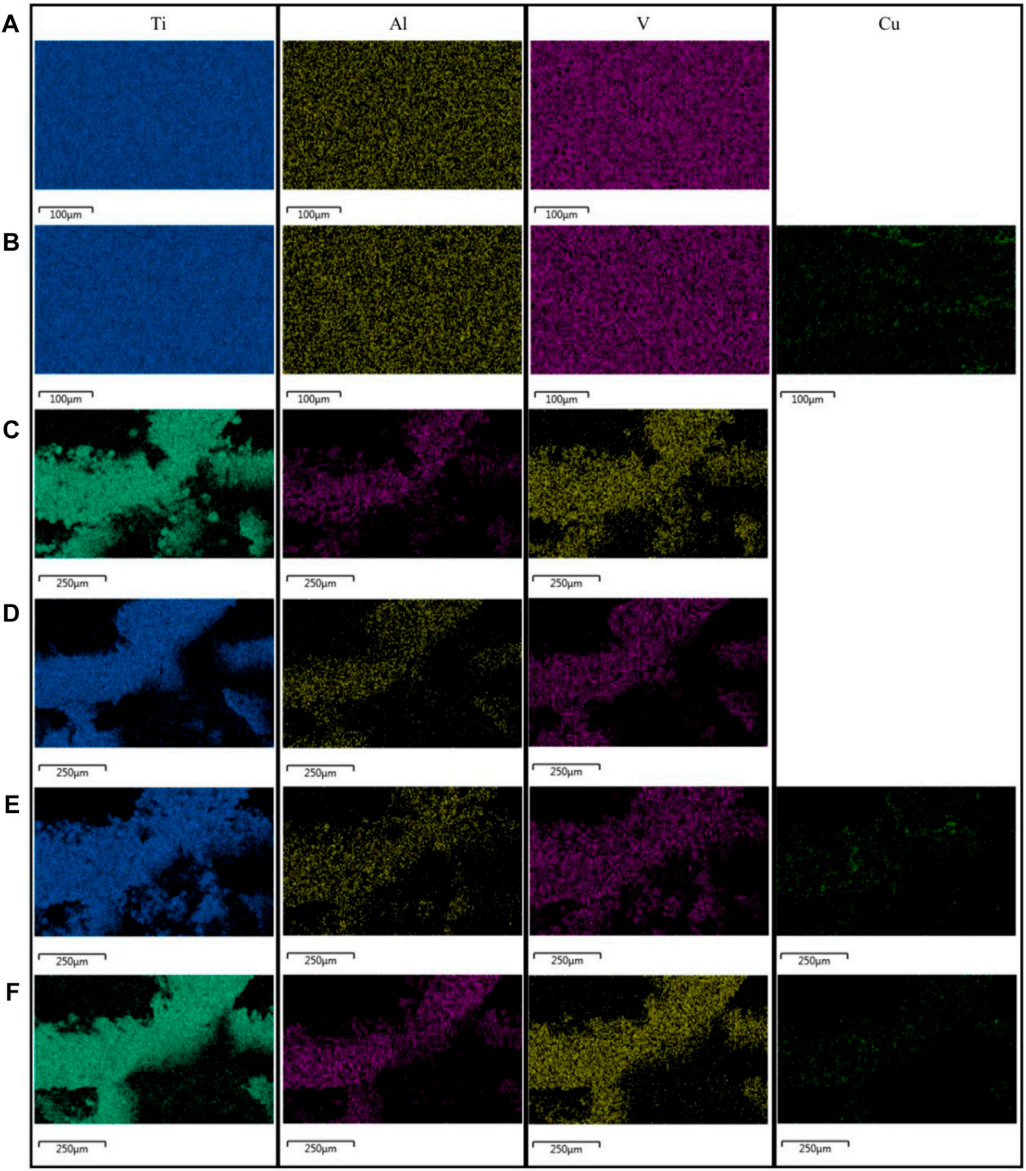
Results of the EDS analysis of the materials: **(A)** TC4; **(B)** TC4-6Cu; **(C)** Porous-TC4; **(D)** Porous-TC4-SAE; **(E)** Porous-TC4-6Cu; **(F)** Porous-TC4-6Cu-SAE (SAE: selective acid etching).

The EDS analysis showed a uniform distribution of elements across all materials (Figure 3). Copper-containing materials detected the presence of copper elements, which were evenly distributed.

### 3.2 Morphological changes in adherent P. gingivalis

**FIGURE 4:**
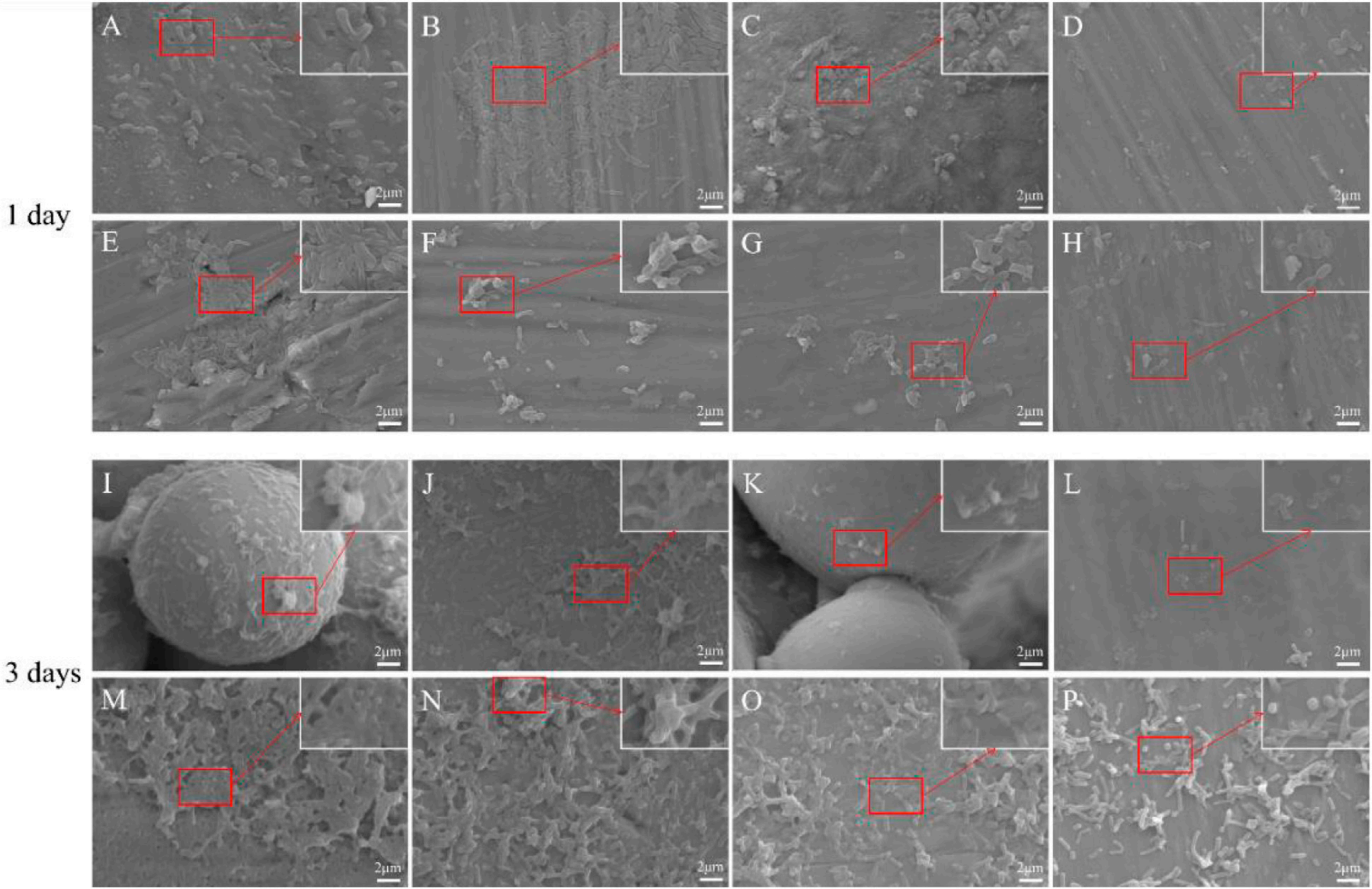
SEM images showing the morphology of bacteria on the materials at 1 and 3 days: **(A,I)** Porous-TC4; **(B,J)** Porous-TC4-SAE; **(C,K)** Porous-TC4-6Cu; **(D,L)** Porous-TC4-6Cu-SAE; **(E,M)** TC4; **(F,N)** TC4-SAE; **(G,O)** TC4-6Cu; **(H,P)** TC4-6Cu-SAE (SAE: selective acid etching).

Morphological changes of *P. gingivalis* adhering to the materials were observed using SEM. The number of *P. gingivalis* on the surface of all copper-containing materials was significantly low (Figure 4). In addition, there was a significant change in the morphology of adherent *P. gingivalis*. On the first day, whether the solid and porous TC4 were acid etched or not, the number of bacteria on the surface of the materials was high, and they densely adhered to the materials. In addition, bacteria appeared in a standard short columnar form. The number of bacteria on the surfaces of solid and porous TC4-6Cu was significantly low. Among them, the number of bacteria on the surface of porous TC4-6Cu after acid etching treatment was the smallest. The bacteria fragmented, atrophied and formed irregular shapes. The number of bacteria on the surfaces of all TC4 materials was significantly higher on the third day than on the first day. The bacteria were stacked and grew layer by layer, densely adhering to the surface of the materials. The number of bacteria on solid TC4-6Cu was less than that of TC4, in which most bacteria were deformed. The un-melted powder was observed on the porous TC4-6Cu without acid etching, and some bacteria adhered to the spherical powder. The spherical powder was removed from the acid-etched materials. Biofilms were significantly low, while the remaining bacteria were wrinkled and deformed.

The SEM results showed that TC4 alloy had no antibacterial properties. The porous TC4-6Cu had the strongest antibacterial properties after acid etching treatment to remove the spherical powder, which could cause bacterial fragmentation and deformation, effectively hindering bacterial adhesion, and bacterial biofilm formation on the material.

### 3.3 Antimicrobial properties of the materials against adherent and planktonic bacteria

**FIGURE 5:**
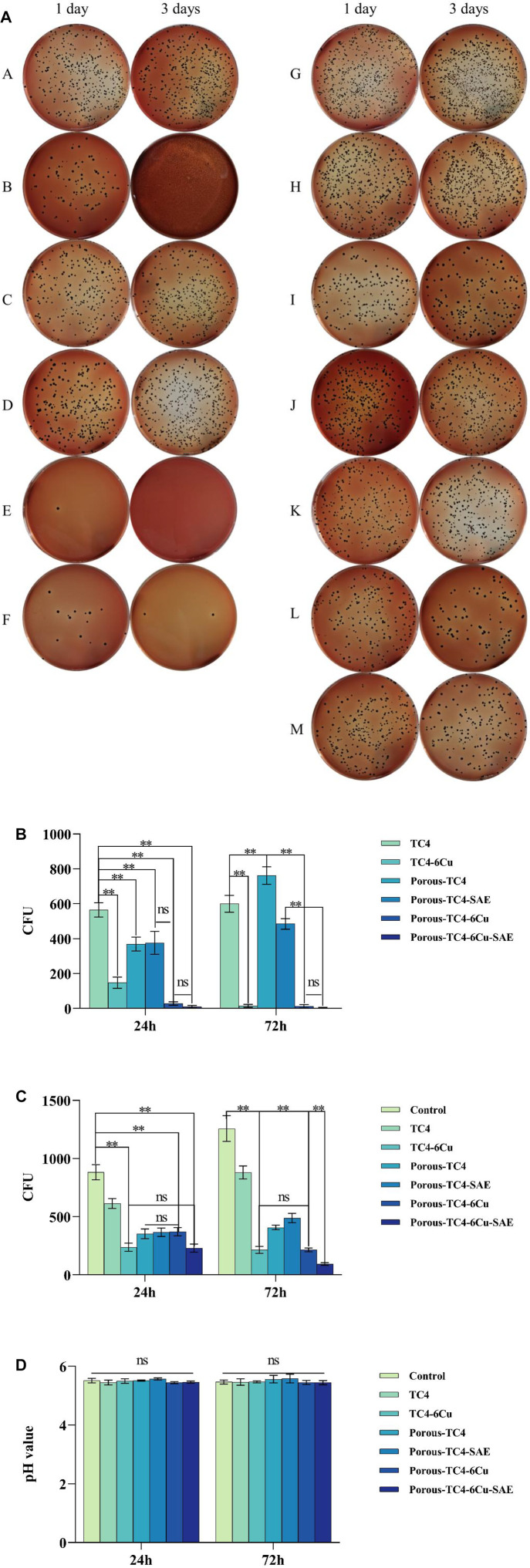
The antimicrobial activity of the materials against adherent and planktonic bacteria: **(A)** adherent (two columns on the left) and planktonic (two columns on the right) bacteria CFUs of the materials at 1 and 3 days: **(A,H)** TC4; **(B,I)** TC4-6Cu; **(C,J)** Porous-TC4-SAE; **(D,K)** Porous-TC4; **(E,L)** Porous-TC4-6Cu-SAE; **(F,M)** Porous-TC4-6Cu; **(G)** Blank control; **(b)** the number of adherent bacteria CFUs; **(c)** the number of planktonic bacteria CFUs; **(d)** pH values of different liquid media at 1 and 3 days (**means *p* < 0.01; ns: not statistically significant; SAE: selective acid etching).

Figure 5 shows the adherent and planktonic colonies of *P. gingivalis* after co-culturing with materials and the pH value of liquid BHI medium. As shown in Figure 5A, the two columns on the left are the adherent colonies, while the two columns on the right are the planktonic colonies. The number of adherent and planktonic bacteria colonies of all TC4 increased over time. Therefore, the antibacterial performance of all TC4 materials was poor, and the effect of acid etching treatment on the antibacterial properties of the materials was negligible. On the third day, all copper-containing materials had a low number of colonies. Porous TC4-6Cu has stronger antibacterial properties than solid copper-containing materials. After acid etching treatment, the porous TC4-6Cu had the lowest number of colonies and the strongest antibacterial performance. The antibacterial rates of the material against adherent bacteria and planktonic bacteria at 24 h were 98.05% and 73.92%, respectively (Figure 5B). No significant antibacterial effect was observed in all materials on planktonic bacteria (Figures 5A,C). Only copper-containing materials showed weak antibacterial capacity, and the trend was similar for antibacterial performance against adherent bacteria. Figure 5D shows that the presence or absence of samples did not affect the pH value of the BHI liquid medium. No significant difference was observed in the pH value of the solution.

Summarily, after acid etching treatment, the porous TC4-6Cu had the strongest antibacterial effect on adhering bacteria. All copper-containing materials had antibacterial activity against planktonic bacteria. However, the effect of the copper-containing materials was not statistically significant.

### 3.4 Quantitative determination of bacterial biofilm

**FIGURE 6:**
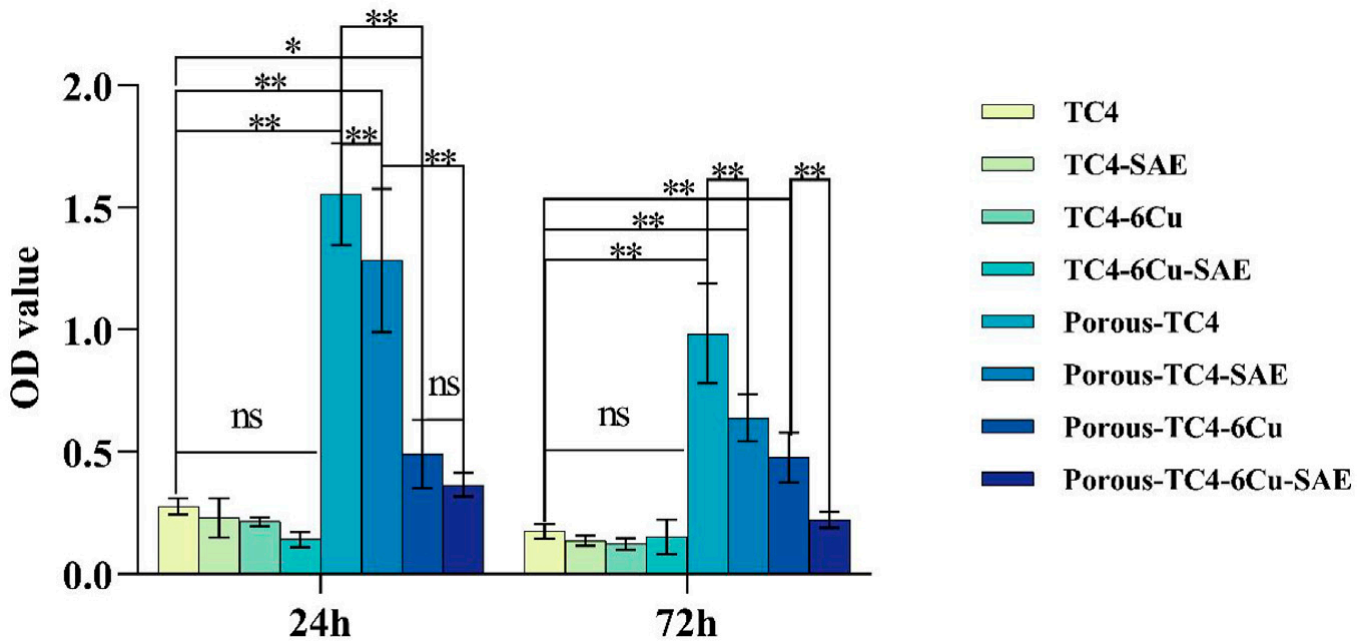
Crystal violet staining results of the materials at 1 and 3 days (*means *p* < 0.05 and **means *p* < 0.01; ns: not statistically significant; SAE: selective acid etching).

The biofilms of *P. gingivalis* on materials were quantified using Crystal violet staining. No statistically significant difference was observed in biofilm amounts for all solid TC4 materials at the two-time points (Figure 6). The amount of biofilm was lower in the solid TC4-6Cu than that in the solid TC4. Nonetheless, acid etching treatment had little effect on the anti-biofilm effect of solid materials. After acid etching treatment, bacterial biofilm was significantly lower on porous TC4-6Cu than on porous TC4 alloy. The biofilm amount was significantly lower on the third day than on the first, corroborating with SEM and colony counting experimental results. Therefore, bacterial biofilm formation was inhibited by porous TC4-6Cu after acid etching treatment.

### 3.5 Activity assay of adherent bacteria

**FIGURE 7:**
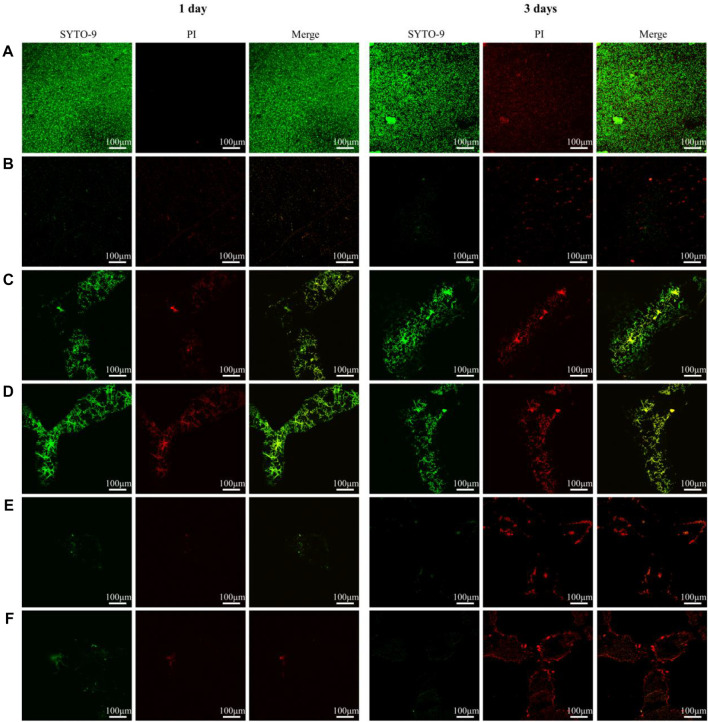
Live/dead staining observation of adherent bacterial biofilm on samples: **(A)** TC4; **(B)** TC4-6Cu; **(C)** Porous-TC4; **(D)** Porous-TC4-SAE; **(E)** Porous-TC4-6Cu; **(F)** Porous-TC4-6Cu-SAE (SAE: selective acid etching).

Figure 7 shows the live-dead staining results of determining adhesion bacterial activity on the materials. At both time points, several viable bacteria were detected on the surfaces of all TC4 alloys. Dead bacteria were few. Over time, the number of live bacteria increased and gathered into clumps. The live bacteria were significantly lower at the two-time points in the copper-containing materials than that in the TC4 alloy. In contrast, dead bacteria were significantly high on the third day. The number of dead bacteria on the surface of the porous TC4-6Cu treated by acid etching was more than that of the non-acid etched porous TC4-6Cu and were densely distributed and partially aggregated into clumps. The above results indicate that the porous TC4-6Cu treated with acid etching could significantly inhibit bacterial biofilm formation and effectively kill bacteria adhering to the surface.

### 3.6 A qualitative study of oxidative stress in bacteria

**FIGURE 8:**
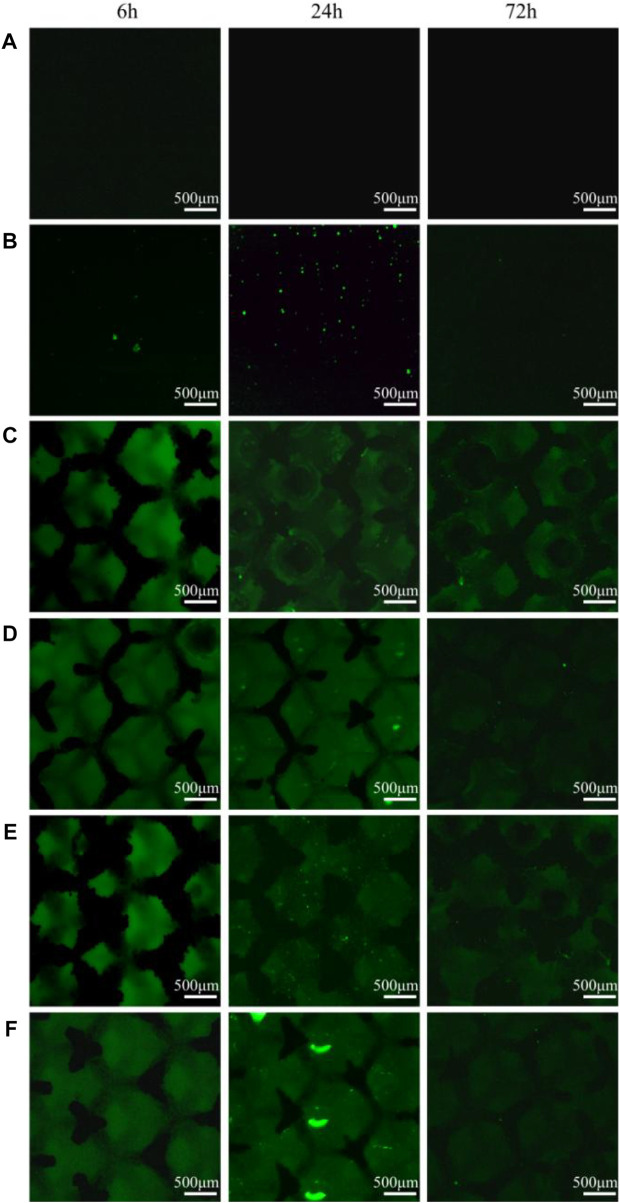
Intracellular ROS staining results at different co-culture time points: **(A)** TC4; **(B)** TC4-6Cu; **(C)** Porous-TC4; **(D)** Porous-TC4-SAE; **(E)** Porous-TC4-6Cu; **(F)** Porous-TC4-6Cu-SAE (SAE: selective acid etching).

Bacterial intracellular ROS staining was performed at 3-time points to determine whether ROS was involved in the antimicrobial process of the materials. As shown in Figure 8, the ROS signals of all TC4 materials were negative, whereas the solid TC4-6Cu had the strongest ROS signal at 24 h. The porous TC4-6Cu expressed the strongest ROS signal at 24 h after acid etching. At the same time, the green fluorescence generated by ROS was densely distributed on the three-dimensional structural surface of the porous material. The signal intensity of copper-containing materials was weakened at 72 h. Only a few dispersed ROS-positive spots were detected. These results confirm that copper-containing materials induce the production of ROS in cells of *P. gingivalis*. For instance, after acid etching treatment, the porous TC4-6Cu stimulated the bacteria to produce the largest amount of ROS and the strongest antibacterial properties.

### 3.7 A quantitative study of bacterial oxidative stress

**FIGURE 9:**
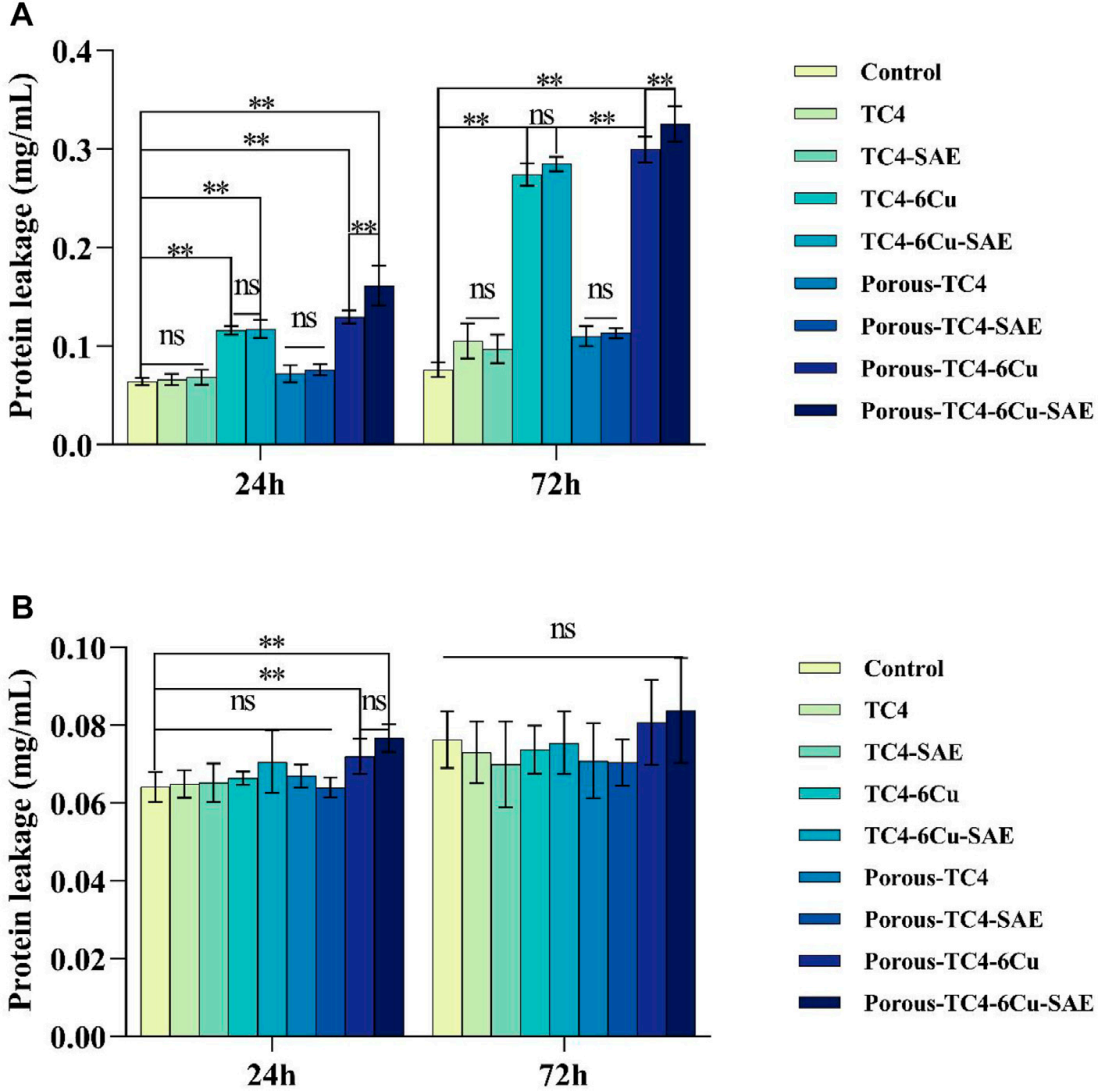
The integrity of bacterial cell membrane was explored based on the concentration of leaked protein: **(A)** the concentration of leaked protein from adherent bacteria on the materials compared to the blank control; **(B)** the concentration of leaked protein from planktonic bacteria in the liquid media compared to the blank control (**means *p* < 0.01; ns: not statistically significant; SAE: selective acid etching).

By detecting the amount of protein leakage caused by ROS, the physiological characteristics of bacteria were investigated after co-culturing with the materials. Adherent and planktonic bacteria in culture media were collected after 1 and 3 days of co-culture with the materials, respectively. The amount of protein leakage was quantitatively investigated. As shown in Figure 9A, among the adherent bacteria on the materials, no significant difference was observed in the amount of bacterial protein leakage between all TC4 materials and the blank control group. The concentration of protein leaked by bacteria was higher on copper-containing materials than on the blank control group, which was proportional to time. No statistically significant difference was noted between acid-etched solid copper-containing materials and non-acid-etched solid copper-containing materials. A statistically significant difference existed between the acid-etched porous copper-containing materials and non-acid-etched porous copper-containing materials. The concentration of protein leaked by bacteria on porous TC4-6Cu after acid etching was the highest. Figure 9B shows the protein concentration of planktonic bacterial leakage in a liquid BHI medium. The results showed that the leakage concentration of bacterial protein in the medium immersed in porous copper-containing materials was higher at 24 h. In addition, no statistical difference was observed in the rest of the data. In summary, the porous copper-containing materials had the strongest capacity to induce oxidative stress in bacteria after pickling. The antibacterial ability of the materials for planktonic bacteria in the medium was weak, consistent with the plate antimicrobial counting experiment.

### 3.8 MIC and MBC values

**FIGURE 10:**
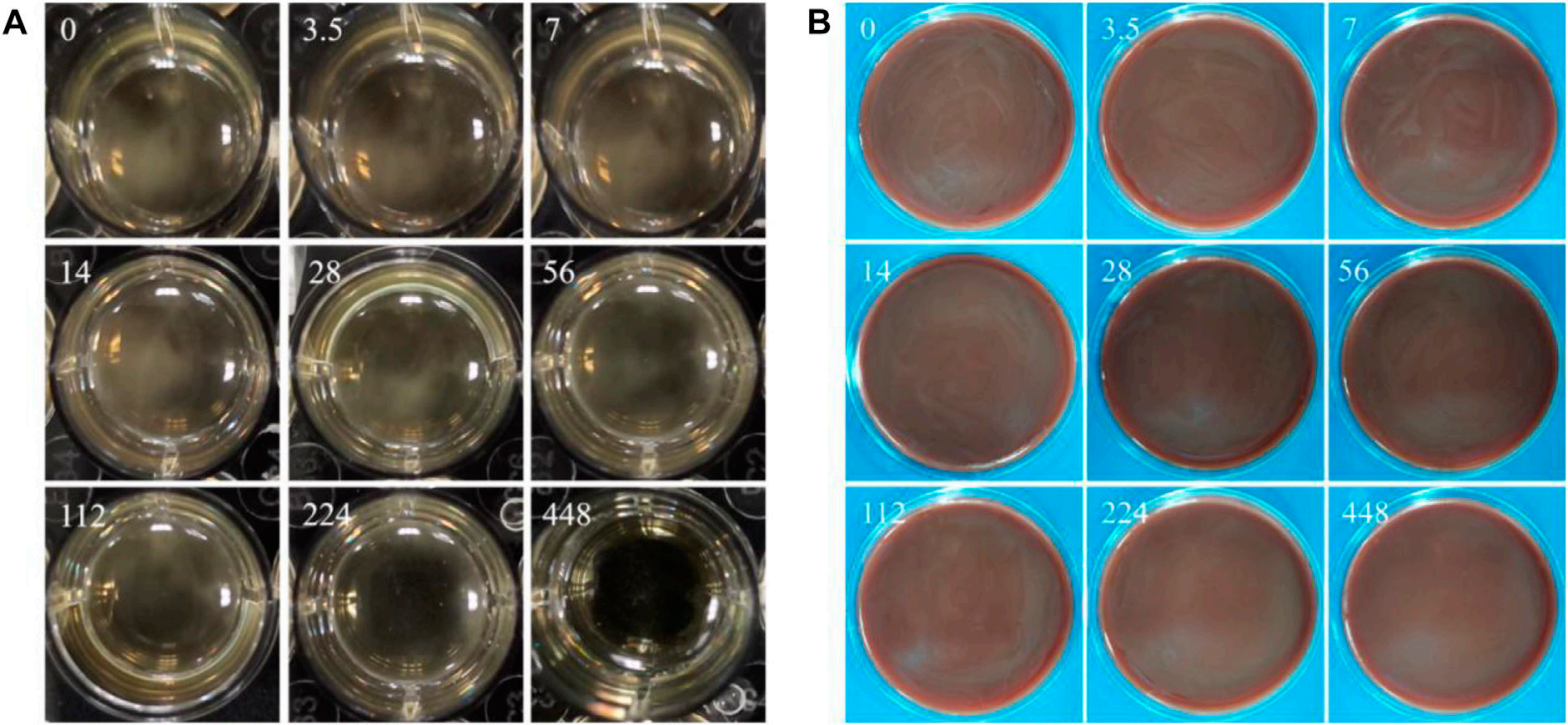
**(A)** Macroscopic images showing the bacteria in liquid BHI medium with gradient copper ion concentrations; **(B)** visible bacterial clumps on BHI-S blood agar plates (numbers (mg/L) in images representing the concentration of copper ions).

The MIC value is the minimum copper ion concentration in the liquid medium without visual bacterial masses. The MBC value is the minimum copper ion concentration of non-visually visible bacterial clumps on solid media. Figure 10A shows that the MIC value of copper ions for *P. gingivalis* is 448 ppm. Figure 10B shows that the MBC value of copper ions for *P. gingivalis* is greater than 448 ppm.

## 4 Discussion

In dental implant surgery, *P. gingivalis* promotes the occurrence and development of peri-implantitis (Lunar Silva and Cascales, 2021). *Porphyromonas gingivalis* can adhere to implant surfaces and oral tissues aggregating to form bacterial biofilm, resulting in infection (Miller and Scott, 2021). Dental implants have a 5-year survival rate of 97.2%, a 10-year survival rate of 95.2%, and a 5-year soft tissue complication rate of 7.1% (Jung et al., 2012). Thus, there is a need for comprehensive research on implant materials with antibacterial functions to prevent the occurrence of peri-implantitis. Copper is an important trace element in the human body with powerful antibacterial properties (Liu et al., 2022). Unlike other antibacterial metal elements, copper is safer and more reliable. Scholars have developed integral copper-containing materials (Ren et al., 2021). However, few studies have explored the preparation of 3D-printed porous titanium-containing alloys using additive manufacturing technology. To investigate the antibacterial effect of copper on *P. gingivalis*, a 3D-printed porous copper-containing titanium alloy was prepared through additive manufacturing technology. Previous studies have shown that addition of 5 wt% (Li et al., 2016) or 10 wt% (Wang et al., 2019) copper to the materials results in strong antibacterial effect and biocompatibility. Therefore, we prepared a 3D printed porous copper-containing titanium alloy with a copper content of 6 wt%. Furthermore, a series of experiments were conducted to investigate the antibacterial ability of the material, the antibacterial mechanism of copper, the effect of selective acid etching treatment on the antibacterial performance and antibacterial mechanism of the material.

The emerging 3D printing technology combines material science and biomedical engineering, revolutionizing the field of tissue engineering (Liang et al., 2022). Because the essence of additive manufacturing technology is the layer-by-layer melting of metal powder, it is impossible to avoid the presence of un-melted powder particles on the surface of the molded material. These particles are unstable and can easily peel off during and after implantation surgery because of fretting and abrasion to form wear particles, causing a macrophage-mediated inflammatory response and peri-implant inflammation, resulting in implant sterility, bacteriological loosening, and eventual failure. Synchrotron radiation X-ray fluorescence spectroscopy (SRXRF) and polarized light microscopy (PLM) were applied to analyze the incidence of different elements (Ca, P, Ti, Fe) in bone and soft tissue samples from patients with peri-implantitis. SRXRF revealed the presence of titanium (Ti), PLM explored the existence of metal particles in the soft tissues surrounding the implant, and lymphocytes were detected in samples with increased titanium concentrations. These results showed that titanium wear particles may induce a specific immune response, causing peri-implantitis (Fretwurst et al., 2016). Daniel G. Olmedo et al. discovered that patients developing peri-implant inflammation after titanium dental implant surgery showed higher concentrations of titanium particles in the surrounding tissues (Olmedo et al., 2013). Hui Liu et al. treated the titanium-copper alloy with HCl/H_2_SO_4_ acid etching, and found increased copper ions release on the surface of the treated material, effectively killing *Staphylococcus aureus* (Liu et al., 2020b). Elsewhere, Rui Liu et al. found a higher release of copper ions from the treated Ti-Cu material at various time points than that of the untreated material, indicating that acid etching treatment can promote the release of copper ions from the material (Liu et al., 2020a). Therefore, a series of experiments were performed to study the effects of the antibacterial mechanism of copper and selective acid etching on the antibacterial properties of materials.

Micro-CT and scanning electron microscopy images showed that selective acid etching did not significantly influence the structure and pore size of the material. Material characterization revealed uniform copper distribution on the alloy surface. Scanning electron microscopy, laser confocal microscopy, and crystal violet tests were used to comprehensively assessing the anti-biofilm capacity of the material against *P. gingivalis*. SEM and CLSM images showed that the porous TC4-6Cu after acid etching caused a strong bactericidal effect on adherent bacteria, and effectively inhibited the adhesion aggregation of *P. gingivalis*. The quantitative detection results of crystal violet demonstrated that copper-containing materials reduced biofilm formation. These results were similar to that of the TC4-5Cu/TC4 alloy which was reported to inhibit the formation of *Streptococcus mutans* biofilm. This demonstrates that the copper-containing titanium alloy has strong bactericidal and anti-biofilm capacity against adherent bacteria (Dong-Yang Fan et al., 2021). Notably, the MIC value and MBC value of copper ions for *P. gingivalis* increased. There was no statistically significant difference in pH change in the BHI liquid medium co-cultured with the material and bacteria at either time point, indicating that the antibacterial ability of the material to the planktonic bacteria in the medium was primarily induced by oxidative stress, rather than the change of pH value or copper ion release in the material (Wang et al., 2017).

Disruption of the balance between ROS production and bacterial antioxidant defense mechanisms causes the accumulation of hydroxylated highly active ingredients, including HCI, H_2_O_2_, and O^2-^. This gradual accumulation of oxidative stress disrupts the bacterial cell membrane increasing its permeability, damaging the bacterial lipids, proteins, and DNA, eventually causing bacterial death (Li et al., 2016). To further investigate the antimicrobial mechanism of materials against bacteria, the following studies were conducted. ROS in bacterial cells can oxidize the 2′, 7′-dichlorodihydrofluorescein-acetoacetate probe (DCFH-DA) to DCF and exhibit strong fluorescence. Therefore, DCFH-DA was used to qualitatively measure ROS levels in cells (Wu et al., 2020). The qualitative and quantitative outcomes of ROS revealed that copper-containing materials increased the level of endogenous oxidative stress in bacteria, improved the permeability of bacterial cell membranes, and promoted protein leakage in bacterial cells. Eventually, this excessively oxidized environment caused bacterial death. Similar to the findings reported in the literature, Li et al. investigated the effect of copper-induced ROS by testing the antibacterial effect of Ti6Al4V-5Cu alloy on *Staphylococcus aureus*. Consequently, copper ions were released from the alloy, which improved the overall permeability of the plasma membrane, resulted in membrane rupture and subsequent protein leakage within the cell. The research group also investigated the ROS concentrations within the bacteria and discovered significantly increased fluorescence intensity of DCF, a trend that was dominant with prolonged exposure. Guangyu Ren et al. designed a novel Co_0.4_FeCr_0.9_Cu_0.3_ antibacterial high-entropy alloy with antibacterial properties without intricate or rigorous annealing processes. The results of the reactive oxygen species analysis revealed that the copper ion release and the immediate contact with the copper-rich phase had a synergistic effect in improving antibacterial properties (Ren et al., 2022). Additionally, free radicals generated by oxidative stress caused lipid peroxidation of cell membranes by reducing their integrity and fluidity (Li et al., 2016). In this experiment, the surface area of porous materials was much larger than that of solid materials, hence the distribution range of copper elements on their surface was also wider, justifying why the porous TC4-6Cu experimental group exhibited the strongest oxidative stress phenomenon at 24 h (Fazel et al., 2021).

In oral implant surgery, strategies for inhibiting the formation of biofilms of dental implants should be implemented. *Porphyromonas gingivalis* can adhere to and accumulate on the surface of implant materials to form dense biofilm structures. Bacterial adhesion is usually divided into two stages to form a mature biofilm. The first stage is characterized by rapid and reversible initial interaction between the bacterial cell surface and the material surface. The second stage involves specific and non-specific interactions between proteins on the bacterial surface structure and binding molecules on the material, slowly progressing irreversibly (Arciola et al., 2018). The use of acid-etched 3D-printed porous TC4-6Cu with a good antibacterial effect on oral implantation is expected to significantly reduce the peri-implantitis incidence and improve the success rate of implantation. This experiment lays a theoretical foundation for futher application of 3D-printed porous TC4-6Cu alloy in dental implants.

## 5 Conclusion

In conclusion, a 3D-printed porous TC4-6Cu alloy was prepared using selective laser melting technology. The un-melted metal particles on the surface of the material were removed by selective acid etching treatment. The copper element was uniformly distributed on the surface, inducing the strongest resistance to the formation of *P. gingivalis* biofilm. Qualitative and quantitative experiments on oxidative stress demonstrated that 3D printed porous TC4-6Cu alloy induced the occurrence of intracellular oxidative stress of *P. gingivalis*, causing protein leakage from the bacterial cell membrane, hence killing the bacteria. Based on the above experimental results, 3D printed porous TC4-6Cu alloy has broad prospects in the clinical application of dental implant materials for preventing peri-implantitis.

## Data Availability

The raw data supporting the conclusion of this article will be made available by the authors, without undue reservation.
